# Safety and effect of pipeline flex embolization device for complex unruptured intracranial aneurysms

**DOI:** 10.1038/s41598-023-31638-0

**Published:** 2023-03-20

**Authors:** Shun-Qiang Chen, Li Li, Bu-Lang Gao, Qiao-Wei Wu, Qiu-Ji Shao, Zi-Liang Wang, Kun Zhang, Tian-Xiao Li

**Affiliations:** Henan Provincial People’s Hospital, Zhengzhou University, 7 Weiwu Road, Zhengzhou, 450000 Henan Province China

**Keywords:** Neuroscience, Diseases, Neurology

## Abstract

To investigate the safety and short-term effect of Pipeline Flex devices in the treatment of complex unruptured intracranial aneurysms, a retrospective study was performed for patients with complex unruptured intracranial aneurysms who were treated with the Pipeline Flex embolization device (PED Flex device) combined with or without coiling. The clinical, endovascular, and follow-up data were analyzed. One hundred and thirty-one patients with 159 complex unruptured cerebral aneurysms were treated with the PED Flex device, with 144 Flex devices deployed. Periprocedural complications occurred in four patients, resulting in the complication rate of 3.1%, including ischemic complications in three patients (2.3%) and hemorrhagic complication in one (0.8%). At discharge, the mRS was 0 in 101 (77.1%) patients, 1 in 25 (19.1%), 2 in four (3.1%), and 4 in one (0.8%), with the good prognosis rate (mRS 0–2) of 99.2%. Clinical follow-up was carried out in 87 (66.4%) patients 3–42 months after the procedure, with the mRS of 0 in 78 (89.7%), 1 in five (5.7%), 2 in three (3.4%), and 4 in one (1.1%). No significant (*P* = 0.16) difference existed in the mRS at discharge compared with that at clinical follow-up. Angiographic follow-up was performed in 61 (46.7%) patients with 80 (50.3%) aneurysms at 3–40 months, with the OKM grade of D in 57 (71.3%) aneurysms, C in eight (10%), and B in 15 (18.8%). Asymptomatic instent stenosis occurred in four patients (6.6%). In conclusion: The treatment of complex intracranial aneurysms with the Pipeline Flex embolization device may be safe and effective, with a high complete occlusion rate, a decreased complication rate, and a good prognosis rate at medium follow-up.

## Introduction

The range of intracranial aneurysms suitable for endovascular management has been significantly expanded since the advent of flow diverters, and aneurysms previously challenging or difficult for either surgical or endovascular treatment have currently become easily manageable with the flow diverter^1–4^. The use of flow diverters has significantly increased the rate of complete aneurysm occlusion and decreased the rate of periprocedural complications or aneurysm recurrence^1–7^. The Pipeline Embolization Device (PED, Medtronic, Irvine, CA, USA) is one of the flow diverters mostly used for the treatment of cerebral aneurysms, with its safety and efficacy being proved by studies indicating a high complete aneurysm occlusion rate and a low rate of major adverse events^3,5,7^. The PED Classic device is the first-generation of the PED flow diverter approved in 2011, which had been demonstrated to have difficulties in deployment and wall adherence, with increased periprocedural complications, including intracranial hemorrhage, stroke, and arterial dissection^8–11^. The second-generation PED Flex device was designed to resolve the issues related to the first-generation device, with improved resheathing and releasing capability as well as modified pusher wires^1,12^. Improved clinical outcomes have also been reported for the PED Flex device, including reduced technical failure, shortened operating time, and decreased periprocedural complication rates^13–15^. For complex cerebral aneurysms like small, wide-necked, large, giant, dissecting, and fusiform aneurysms, the safety and clinical effect after treatment with the PED Flex device are unknown. Although some individualized studies have reported some short-term outcomes using the PED device in treating complex aneurysms^13,16^, not a single study has been reported to include most complex cerebral aneurysms in one study with the same group of physicians and similarly proficient endovascular embolization skills using the PED Flex device in a hospital with a large volume of patients. It was hypothesized that the PED Flex device acted well in treating complex intracranial aneurysms as the first-general PED device, and patients with complex aneurysms treated in a large-volume hospital by the same group of physicians with the same proficiency in endovascular embolization skills would greatly improve the treatment outcome and decrease the complication rate using the PED Flex device. This study was consequently performed to investigate the safety and efficacy of the second-generation PED Flex in the treatment of complex cerebral aneurysms.

## Materials and methods

This retrospective study was approved by the ethics committee of Henan Provincial People’s hospital, and all patients or their family members had given the signed informed consent to participate. All methods were performed in accordance with the relevant guidelines and regulations. Between February 2018 and September 2019, patients with complex unruptured cerebral aneurysms treated with the PED Flex device were enrolled. The inclusion criteria were patients with complex unruptured intracranial aneurysms confirmed by medical imaging, including tiny (< 3 mm in diameter because of the small size for a clip or coil), large (> 10 mm), giant (> 25 mm), wide-necked (> 4 mm or neck to dome ratio > 1/2), tandem multiple, and blood-blister aneurysms^17^, treated with the PED device for at least one aneurysm in each patient. Patients who did not have the above complex aneurysms or patients with simple (non-complex) aneurysms were excluded from the study.

Three to five days before the endovascular procedure, all patients took dual antiplatelet therapy (clopidogrel 75 mg/d and aspirin 100 mg/d). Then, thromboelastogram was tested to ensure the arachidonic acid inhibition rate > 50%, the adenosine diphosphate inhibition rate > 30%, and the adenosine diphosphate curve maximal amplitude value being controlled at 31–47 mm. The antiplatelet medication was adjusted in the dosage and usage based on the test outcome of the thromboelastogram with the dosage being increased or replacement of clopidogrel by Tegrello (Ticagrelor, 90 mg twice daily). During the endovascular treatment procedure, heparin was administered (50–70 U/kg) intravenously. After endovascular treatment, the dual antiplatelet therapy was continued at the same dosage or with the dosage being adjusted according to the test outcome of the thromboelastogram in all patients for at least six months followed by long-term (over two years) use of aspirin (100 mg/d) or clopidogrel (75 mg/d). At discharge, repeated thromboelastogram was performed for patients who did not have good control of the arachidonic acid inhibition rate and adenosine diphosphate inhibition rate, and Tegrello (Ticagrelor, 90 mg twice daily) was used to replace clopidogrel for those who did not have good control of the above test.

The endovascular procedure was performed under general anesthesia. After insertion of a long arterial sheath in the right femoral artery, a 6F Navien (ev3/Covidien, USA) guiding catheter was introduced to the parent artery proximal to the aneurysm before three-dimensional angiography. After the aneurysm and parent artery were measured, an appropriate PED Flex device was selected for deployment. The principle of PED device selection was based on the diameter of the PED device which should be as close to the diameter of the parent artery as possible while ensuring the adherence of the device. The diameter of the PED device could be 0.1–0.25 mm greater than that of the parent artery, and the length should cover the aneurysm neck with a stable anchoring distance at both ends, generally 5–10 mm. For tandem aneurysms or several aneurysms located closely together, a PED device should be selected long enough to cover all these aneurysms, and the biggest aneurysm was embolized with coils. In addition to deployment of the PED Flex device, coiling was performed in symptomatic (mass effect or other neurological symptoms), irregular, large or giant cerebral aneurysms or aneurysms with a daughter dome to promote quick thrombosis of the aneurysm cavity and prevent possible rupture. Coiling was performed with the micro-catheter being jailed between the PED device and the parent arterial wall in all cases because of the dense mesh of the PED device. Under road map guidance, a micro-guidewire (Synchro, Stryker, USA) was used to guide the stent delivery system (Marksman, Medtronic, USA) to the appropriate location for deployment. In coiling, an embolizing catheter (Echelon 10, Medtronic, USA) was navigated into the aneurysm sac for coil embolization. If the PED Flex device was not well adherent to the arterial wall on repeated two-dimensional or three-dimensional digital subtraction angioraphy, a micro-guidewire together with a microcatheter was used to “massage” the Flex device, or a balloon catheter was used to expand the device for good wall adherence. After embolization, digital subtraction angiography was performed to check the embolization and wall adherence of the device. Immediate DYNA CT (computed tomography) was conducted to exclude intracranial hemorrhage.

The clinical effect was evaluated immediately after embolization and at follow-up using the modified Rankin scale (mRS) score, with the mRS ≤ 2 being defined as good prognosis. Six months later, angiographic follow-up was performed to check the aneurysm occlusion status with the O’Kelly-Marotta (OKM) grading system^18^, with the aneurysm filling being graded as: A—complete (> 95%), B—incomplete (5%-95%), C—neck remnant (< 5%), and D—no filling (0%).

### Statistical analysis

Statistical analysis was performed with the SPSS 19.0 software (IBM, Chicago, IL, USA). Measurement data were presented as mean ± standard deviation if in normal distribution and tested with the student t test or median and interquartile range if in skew distribution and tested with the Chi square test. Enumeration data were presented as numbers and percentages and tested with the Chi square test. The significant *P* value was set at < 0.05.

## Results

One hundred and thirty-one patients with 159 complex aneurysms were enrolled, including 43 male and 88 female patients, with an age range of 27–78 (mean 54.2 ± 9.7) and the aneurysmal maximal diameter of 7.5 ± 3.4 mm (Table 1). The location of aneurysm was the internal carotid artery in 135 (84.9%) aneurysms, including the cavernous segment in 12 (7.5%), clinoid segment in 15 (9.4%), ophthalmic segment in 76 (47.8%), and posterior communicating segment in 32 (20.1%), middle cerebral artery in 6 (3.8%), vertebral artery intracranial segment in 15 (9.4%), and basilar artery trunk in 3 (1.9%). One hundred and seven (82.4%) patients had one aneurysm each, and 24 (17.6%) had multiple aneurysms.

**Table 1:** Demography and clinical data.

Variables		Data
Patients	F/M	88/43
	Age (y)	27–78 (54.2 ± 9.7)
	Weight (kg)	56–85 (64.2 ± 11.2)
	BMI	22.1–28.6 (25.3 ± 7.4)
	Diabetes mellitus (n)	16 (12.21%)
	Heart disease (n)	21 (16.03%)
	Hyperlipidemia (n)	13 (9.92%)
	Hypertension (n)	26 (19.85%)
	Smoking (n)	37 (28.24%)
	Alcohol abuse (n)	32 (24.43%)
Aneurysm location (n,%)	ICA Cavernous segment	12 (7.5%)
	ICA Clinoid segment	15 (9.4%)
	ICA ophthalmic segment	76 (47.8%)
	ICA Pcom segment	32 (20.1%)
	MCA M1 segment	6 (3.8%)
	Vertebral V4 segment	15 (9.4%)
	Basilar artery trunk	3 (1.9%)
No. of aneurysms (n,%)	Patients with one aneurysm each	107 (82.4%)
	Patients with multiple aneurysms each	24 (17.6%)
Aneurysm size (mm)		7.5 ± 3.4

ICA, internal carotid artery; MCA, middle cerebral artery; Pcom, posterior communicating artery.

**Figure 1:**
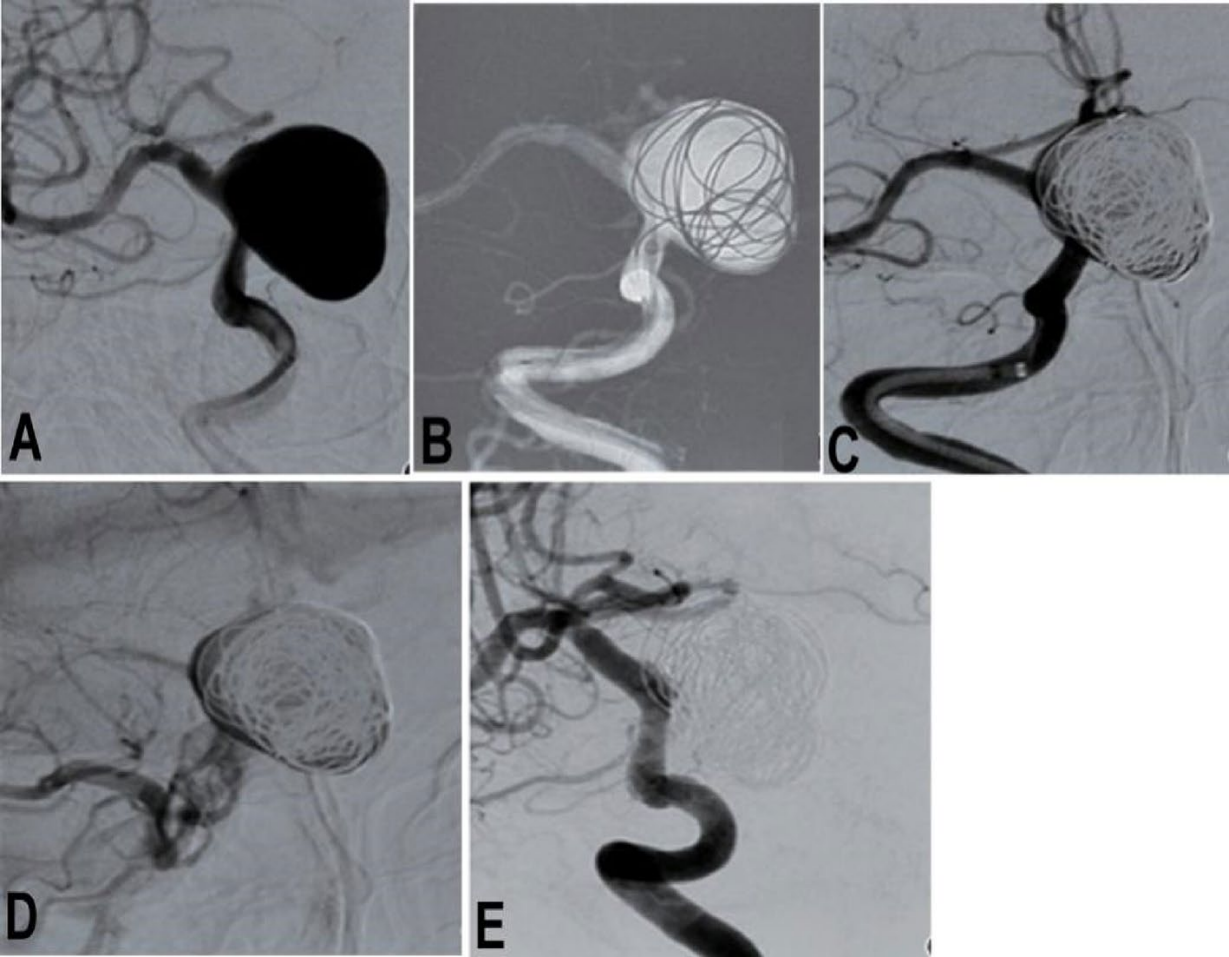
A patient with a giant aneurysm at the right internal carotid artery ophthalmic segment was treated with a Pipeline embolization device combined with coil embolization. (**A**) Before endovascular procedure, the giant aneurysm was shown. (**B**) The aneurysm was firstly partially embolized with coils to promote thrombosis within the aneurysm sac and prevent possible aneurysm rupture before deployment of the Pipeline device. (**C**) After embolization, angiography revealed contrast filling inside the aneurysm sac. (**D**) In the venous phase, the contrast agent within the aneurysm was retained. (**E**) Follow-up angiography 4 months later showed complete occlusion of the aneurysm.

**Figure 2:**
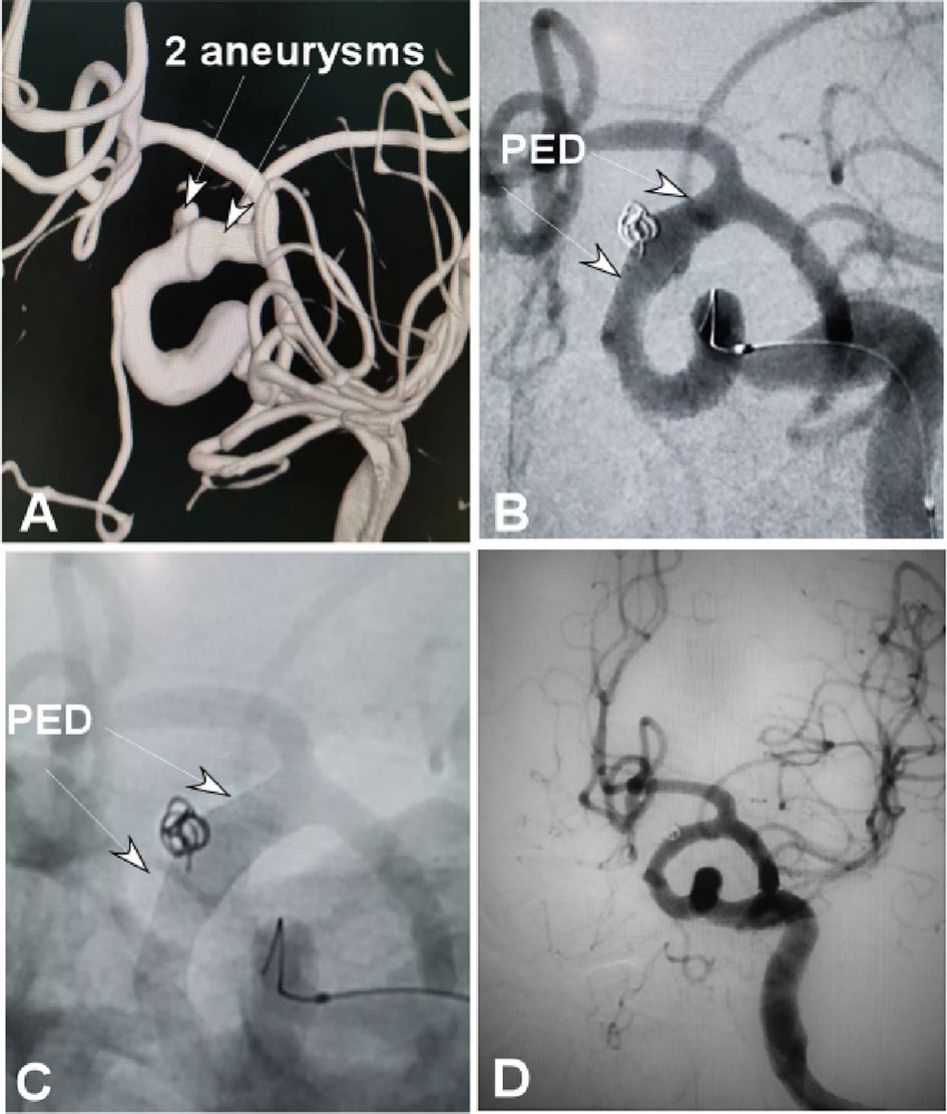
A middle-aged women had headache. (**A**) Three-dimensional imaging revealed two small aneurysms at the C6-7 segments of the left internal carotid artery growing in two different dirrections. (**B**), (**C**). After one Pipeline embolization device (PED) was deployed (arrows), the smaller aneurysm sac was coiled with the microcatheter tip being jailed between the PED and the parent arterial wall, and the small aneurysm sac obtained complete occlusion, with patency of the parent artery. (**D**) Follow-up angiography 6 months later demonstrated complete aneurysm occlusion and patent parent artery.

One hundred and forty-four PED Flex devices were all successfully deployed to treat 159 aneurysms harbored in the 131 patients, with the stenting success rate of 100% and a mean operation time of 123.7 ± 49.1 min (Table 2 and Figs. 1 and 2). A Flex device alone was used in 107 (67.3%) aneurysms while a Flex device combined with coiling was applied to treat 52 (32.7%) aneurysms. All devices had good coverage of the aneurysm neck with good wall adherence and patent parent artery. No patients experienced the “massage technique” or “balloon expansion” technique to make the PED device well adherent to the parent arterial wall. At discharge from the hospital, the mRS was 0 in 101 (77.1%) patients, 1 in 25 (19.1%), 2 in four (3.1%), and 4 in one (0.8%), with the good prognosis rate (mRS 0–2) of 99.2%.

**Table 2:** Endovascular treatment and follow-up.

Stenting procedures	No. of flow diverters deployed	144
	Aneurysms with diverters only (n)	107 (67.3%)
	Aneurysms with diverters and coiling (n)	52 (32.7%)
	Success rate of procedure	100%
	Mean operation time (min)	123.7 ± 49.1
Periprocedural complications	Ischemic	3 (2.3%)
	Hemorrhagic	1 (0.8%)
mRS at discharge	mRS 0 (n,%)	101 (77.1%)
	mRS 1 (n,%)	25 (19.1%)
	mRS 2 (n,%)	4 (3.1%)
	mRS 4 (n,%)	1 (0.8%)
Clinical follow-up	Duration (m)	3–42 (median 28)
	mRS 0 (n,%)	78 (89.7%)
	mRS 1 (n,%)	5 (5.7%)
	mRS 2 (n,%)	3 (3.4%)
	mRS 4 (n,%)	1 (1.1%)
Angiographic follow-up	Duration (m)	3–40 (median 26)
	No. of patients (n,%)	61 (46.7%)
	No. of aneurysms (n,%)	80 (50.3%)
	OKM grade D	57 (71.3%)
	OKM grade C	8 (10%)
	OKM grade B	15 (18.8%)
	Instent stenosis	4 (6.6%)

mRS, modified Rankin Scale; OKM grade, O’Kelly-Marotta grading system. No significant (*P* = 0.16) difference existed in the mRS at discharge compared with that at clinical follow-up.

Periprocedural complications occurred in four patients (3.1%), including ischemic ones in three (2.3%) and hemorrhagic in one (0.8%). No death occurred. In one case treated with a stent “bridging technique” for multiple aneurysms at the ophthalmic and posterior communicating segments of the left internal carotid artery and M1 segment of the left middle cerebral artery, no blood flow was demonstrated through the distal end of the parent artery following deployment of the second Flex device. After application of Tirofiban through intravenous and transcatheter injection, the blood flow was slowly recovered, and a balloon catheter was used to expand the bridging part of the two Flex devices because of poor wall adherence. Five days after the procedure, sudden limb movement dysfunction took place with the right muscle strength of grade 1 and mixed aphasia, and emergency digital subtraction angiography showed parent artery occlusion. Mechanical embolectomy was conducted to restore the patency of the parent artery, and the mRS reached 4 at discharge. In one case with a dissecting aneurysm at the right ophthalmic segment of the internal carotid artery, right limb weakness occurred suddenly on the second day after embolization, with the muscle strength of grade III in the left upper limb and grade IV in the left lower limb. After administration of Tirofiban for two days, the patient was discharged with an mRS of 1. In one case with an aneurysm at the posterior communicating segment of the left internal carotid artery, limb movement dysfunction occurred four hours after embolization, with the right limb muscle strength of grade 0. Emergency digital subtraction angiography revealed occlusion of the parent artery, and mechanical thrombectomy was performed to restore the patency of the artery, resulting in an mRS of 1 at discharge. In one case with an aneurysm at the ophthalmic segment of the right internal carotid artery, the patient experienced sudden headache three days after embolization, computed tomography demonstrated cerebral hemorrhage, mass effect, and herniation. After emergency craniotomy to evacuate the hematoma, the patient was discharged with an mRS of 2.

Clinical follow-up was carried out in 87 (66.4%) patients 3–42 months (median 28) after the procedure, and no new neurological symptoms were detected, with the mRS of 0 in 78 (89.7%), 1 in five (5.7%), 2 in three (3.4%), and 4 in one (1.1%), with the good prognosis (mRS 0–2) rate of 98.9%. One patient with the mRS of 2 at discharge had the mRS turned to 4, with the incidence of worsening mRS of 1.1%. No significant (*P* = 0.16) difference existed in the mRS at discharge compared with that at clinical follow-up. Angiographic follow-up was performed in 61 (46.7%) patients with 80 (50.3%) aneurysms 3–40 months (median 26) after embolization, with the OKM grade of D in 57 (71.3%) aneurysms, C in eight (10%), and B in 15 (18.8%). The complete aneurysm occlusion rate was 71.3% in 57 aneurysms, and no aneurysm recurrence was found. Asymptomatic instent stenosis occurred in four patients (6.6%).

## Discussion

In this study investigating the safety and short-to-medium-term effect of Pipeline Flex devices in the treatment of complex intracranial aneurysms, it was found that the treatment of complex intracranial aneurysms with the Pipeline Flex embolization device is safe and effective, with a significantly decreased complication rate but a high complete occlusion rate at angiographic follow-up two years later.

Abnormal hemodynamics has been proved to be an important factor affecting the initiation, development, and rupture of intracranial aneurysms^19–25^. The stent mesh of the flow diverting devices represented by the PED ranged 0.02–0.05 mm^2^, and the metal coverage of the parent artery after stent deployment reached 30%-50%, three times that of other conventional intracranial stent like the Neuroform stent^26,27^. The flow diverting devices achieved the treatment goal of aneurysms by correcting local abnormal hemodynamics through reconstructing the parent artery without surgery or in-aneurysm coiling or other operations which may puncture the aneurysm wall^28^. With a large number of application of the first-generation PED Classic device, a lot of shortcomings have been found for the Classic device, including difficult delivery and release of the stent, increased procedural complications such as stroke, intracranial hemorrhage and arterial dissection^8–11^, non-retrieval, and poor wall adherence at tortuous arterial segments^29^. The second generation PED Flex device was designed to solve these issues^12^.

In comparing the effect of the PED Classic (n = 252) and Flex (n-316) devices in the treatment of anterior circulation aneurysms, Colby et al. found the good prognosis rate (mRS 0–2) for the PED Flex device reaching 94.9%, a mild complication rate 7.0%, severe complication rate 1.9%, and a mortality rate 0.6%, with the severe complication rate less than that for the PED Classic device (5.6%)^14^. The multi-center study by Brasiliense et al. evaluating the neurological morbidity and mortality rates of the PED Flex at 30 days after embolization^13^ revealed the 30-day neurological morbidity rate of 1.9%, mortality rate 0.5%, intraprocedural complication rate without neurological morbidity of 6.8% (ischemic complication rate of 4.5% and hemorrhagic rate of 2.4%), favorable clinical outcome (mRS 0–2) of 94.4% at discharge and 94.5% at 30 days after embolization. In our study, the neurological complication rate was 3.1%, including the ischemic rate of 2.3% and hemorrhagic rate of 0.8%, and the favorable clinical outcome was 99.2% at discharge and 98.9% at follow-up. Although the incidence of complications is very low, prevention is still needed.

Reasons for the periprocedural ischemic complications may involve insufficient application of antiplatelet therapy, instent stenosis or occlusion, and occlusion of perforating arterial branches^5^. In our study, three cases had acute instent thrombosis. In one case, the preoperative thromboelastogram was not up to the standard, indicating that the risk of thrombosis was high. In two cases with good thromboelastogram, the parent artery was tortuous, resulting in difficult operation of the Flex device and insufficient wall adherence. In order to prevent the ischemic complication events from happening, some measures should be taken, including test of the sensitivity of the antiplatelet medications, application of Tirofiban, and ensurance of good wall adherence of the Flex device. The sensitivity of aspirin and clopidogrel has been proved to be closely related to the ischemic events after PED treatment of intracranial aneurysms, and thromboelastogram test may help reducing the ischemic events^30,31^. For patients with long endovascular treatment time, poor preoperative thromboelastogram test, bad wall adherence, and possible thrombosis formation within the Flex device, Tirofiban should be used both during the procedure and 24–48 h after the procedure because it can reduce the ischemic events without increasing the risk of hemorrhage^32^. Insufficient wall adherence is an independent risk factor affecting ischemic events after deployment of the PED devices, and with accumulation of operation experience, good wall adherence can be achieved with decreased technique-related complications^10^. Moreover, for patients with over 50% stenosis of the parent artery, balloon expansion should be performed to release part of the stenosis so that the Flex device can have good wall adherence after deployment. In poor wall adherence, a microcatheter combined with a micro-guidewire for “massage” of the PED device and balloon expansion of the device may be necessary.

Instent or intravascular stenosis and restenosis may occur in the PED as well as in other intracranial stents used in treating intracranial vascular diseases^16,33^. The deployment of endovascular stents and flow diverters like the PED will result in de-endothelialization and subsequently a strong inflammatory cascade to cause neointimal hyperplasia. The stents and PED as foreign bodies deployed in the cerebral arteries will also cause a series of inflammatory mediators, prothrombotic factors, cytokines and adhesive molecules to accumulate and produce inflammatory reactions in the stent^34^. These resultant neointimal hyperplasia and inflammatory reactions within the deployed PED device or intracranial stents will likely lead to instent stenosis. Nonetheless, after complete endothelialization of the stent or PED, the inflammatory reactions will be significantly decreased or eliminated. This is why sufficient post-treatment antiplatelet therapy should be administrated in a long term so as to prevent instent stenosis before complete endothelialization of the stent or PED deployed in cerebral arteris. Insufficient wall adherence may exacerbate instent stenosis, which is why good wall adherence should be achieved in deploying the PED device.

In our study, one patient experienced intraparenthymal hemorrhage on the ipsilateral side of the aneurysm after embolization, which was probably caused by hypertension in this patient, alteration of the hemodynamics after deployment of the Flex device, and high concentration of the antiplatelet therapy. Furthermore, occlusion of small distal arterial branches by air bubbles and sheded microemboli may lead to cerebral infarction, and recanalization of these arterial branches may cause reperfusion hemorrhage^5^.

Long-term effects of the PED Flex devices in the treatment of intracranial aneurysms have not been reported sufficiently but may have similar effects to those of the PED Classic device. In three large clinical studies on the PED Classic devices for the treatment of intracranial aneurysms, the complete aneurysm occlusion rate reached 75% at six months after embolization^35^. The multicenter study by Brasiliense et al.^13^ investigating the PED Flex device for the treatment of intracranial aneurysms demonstrated a complete aneurysm occlusion rate of 52.1% at 4.8 months of follow-up. In our study, the complete aneurysm occlusion rate reached 71.3% at a median follow-up time of six months. The long-term occlusion rate of intracranial aneurysms treated by the PED devices primarily depends on the intimal hyperplasia to cover the aneurysm neck, and animal experiments have demonstrated that the time for neointima to completely cover the aneurysm neck ranged 3–6 months, which may be affected by the stent mesh, wall adherence, aneurysm location and size, and coiling^36^. The study by Kallmes et al. revealed that the complete aneurysm occlusion rate reached 75%, 85.5%, 93.4%, and 95.2% at six months, one year, three and five years, respectively^35^.

In our study, coils were used to embolize 52 (32.7%) aneurysms in combination with the deployement of a PED. In the treatment of cerebral aneurysms with flow diverters in our center, coiling was performed in symptomatic, irregular, large or giant cerebral aneurysms or aneurysms with a daughter dome to promote fast thrombosis of the aneurysm cavity and prevent possible rupture or re-rupture. As foreign materials, metal coils can erxert additional thrombosis within the aneurysm cavity and activate inflammatory responses to quickly occlude the aneurysm. Moreover, coils within the aneurysm dome can prevent possible herniation of the PED device into the aneurysm cavity. An increased aneurysm occlusion rate has been demonstrated in patients with cerebral aneurysms which had been treated with the PED device plus coils^37^. At six month-follow-up of patients with cerebral aneurysms treated with the PED device with or without coiling, the complete occlusion rate of aneurysms was 85.4% vs 71.7% for aneurysms treated with the PED device plus coils versus the PED device only^37^. Researchers have also showed that among patients with cerebral aneurysms which were treated with the PED device plus coils or the PED device only without additional coiling^38^, no significant differences were found in adjusted regression analyses between patients treated with PED with and without coils in hemorrhagic or thromboembolic events, residual flow on follow up angiography, aneurysm occlusion rate, or functional outcome. This study^38^ indicates that treatment of cerebral aneurysms with combining PED and coiling is safe and efficient with no increased complications when compared to PED embolization alone.

Although some individual studies have reported some similar outcomes, our study included all complex aneurysms in one study and reported the treatment outcome as well as follow-up ones because of the large volume of patients in our hospital. Our study contributes to a better understanding of use of the PED device in treating complex intracranial aneurysms in one large medical center with the same group of physicians in the treatment of the cerebral aneurysms. Because of the same group of physicians with similar proficiency of skills in the treatment of these patients, the heterogeneity may be greatly reduced, and the efficiency of treatment will be increased with decreased complication rates. The median time is 26 in imaging follow-up and 28 months in clinical follow-up, which is longer than most of other studies. Despite this, our study had some limitations including the retrospective and one-center study nature, a small cohort of patients, Chinese patients enrolled only, no control, and no randomization, which may all affect the generalization of the outcomes. Future randomized, controlled, multicenter clinical trials will have to be performed to resolve all these issues for better outcomes.

In summary, the treatment of complex intracranial aneurysms with the Pipeline Flex embolization device may be safe and effective, with a high complete occlusion rate and a good prognosis rate at six-month follow-up.

## Data Availability

The datasets generated and/or analyzed during the current study are not publicly available because the medical data are owned by the hospital and are not allowed to make publically available but are available from the corresponding author on reasonable request.
